# Viscous Flow of Supercooled Liquid in a Zr-Based Bulk Metallic Glass Synthesized by Additive Manufacturing

**DOI:** 10.3390/ma13173803

**Published:** 2020-08-28

**Authors:** Konrad Kosiba, Liang Deng, Sergio Scudino

**Affiliations:** Leibniz IFW Dresden, Institute for Complex Materials, Helmholtzstrasse 20, 01069 Dresden, Germany; l.deng@ifw-dresden.de

**Keywords:** laser powder bed fusion, additive manufacturing, bulk metallic glass, viscosity, thermoplastic forming

## Abstract

The constraint in sample size imposed by the critical cooling rate necessary for glass formation using conventional casting techniques is possibly the most critical limitation for the extensive use of bulk metallic glasses (BMGs) in structural applications. This drawback has been recently overcome by processing glass-forming systems via additive manufacturing, finally enabling the synthesis of BMGs with no size limitation. Although processing by additive manufacturing allows fabricating BMG objects with virtually no shape limitation, thermoplastic forming of additively manufactured BMGs may be necessary for materials optimization. Thermoplastic forming of BMGs is carried out above the glass transition temperature, where these materials behave as highly viscous liquids; the analysis of the viscosity is thus of primary importance. In this work, the temperature dependence of viscosity of the Zr_52.5_Cu_17.9_Ni_14.6_Al_10_Ti_5_ metallic glass fabricated by casting and laser powder bed fusion (LPBF) is investigated. We observed minor differences in the viscous flow of the specimens fabricated by the different techniques that can be ascribed to the higher porosity of the LPBF metallic glass. Nevertheless, the present results reveal a similar overall variation of viscosity in the cast and LPBF materials, which offers the opportunity to shape additively manufactured BMGs using already developed thermoplastic forming techniques.

## 1. Introduction

If a liquid is cooled sufficiently fast, crystallization can be kinetically suppressed, and a glass is formed [1,2]. Compared to oxide glasses, the cooling rates necessary for glass formation in bulk metallic glasses (BMGs) using casting are significantly higher, imposing size restrictions known in the literature as critical casting dimension [1]. The typical critical dimension of good glass-forming alloys can range from several millimeters to more than one centimeter [3,4]. This size limitation is one major shortcoming that obstructs potential applications of BMGs [3]. Nowadays, additive manufacturing (AM) techniques, such as laser powder bed fusion (LPBF), provide the solution for overcoming this deficiency [5]. Since only small powder volumes are consecutively melted, high cooling rates (10^4^–10^6^ K/s) are inherent to LPBF [6], which generally allows to process a wide variety of metallic glass forming alloys. The resulting BMGs are fabricated “layer-by-layer” with sheer unlimited geometrical freedom [5,7,8]. As a result, a variety of glass-forming compositions, including Al-, Fe-, Ti-, and Zr-based alloys [5,7,8,9,10,11,12,13], have been successfully processed via LPBF in order to synthesize BMGs.

The high freedom to manufacture complex-shaped components by LPBF and other AM techniques comes at a cost, namely the processing control. Multiple processing parameters must be correctly selected to fabricate not only amorphous, but also highly dense BMGs, since the presence of pores and cracks, as well as the formation of crystalline phases, must be avoided. In order to synthesize highly dense BMGs (>98% density [9]), extensive parameter studies have been carried out, particularly for Zr-based alloys, because of their good glass-forming ability [8,9,13,14].

Owing to their high strength, BMGs are considered as potential structural materials. Aside from the microstructure, the mechanical properties of additively manufactured BMGs at room temperature have been and still are the focus of intense research. For example, the mechanical response of additively manufactured BMGs was investigated when subjected to uniaxial compressive loading [8,9,13,15,16] and bending [5,15]. Owing to residual porosity, which acts as a stress concentrator, BMGs fabricated via LPBF yield at lower stresses, show less plastic deformability, and have a lower fatigue threshold [15] than their amorphous counterparts prepared by conventional copper mold casting. During LPBF processing, Zr-based glass-forming alloys are enriched in oxygen so that the resulting BMGs show a distinctly lower fracture toughness than cast BMGs [5,15,16]. By contrast, their wear and corrosion resistances are similar [8,17].

Compared to room-temperature properties of additively manufactured BMGs, little attention has been paid to their structure and resulting properties at elevated temperatures. Only the relaxation behavior was investigated recently for Zr_52.5_Cu_17.9_Ni_14.6_Al_10_Ti_5_ [9] and Zr_59.3_Cu_28.8_Nb_1.5_Al_10.4_ [15] LPBF BMGs. Owing to the high cooling rates effective during LPBF [6], the corresponding BMGs show a lower atomic packing density readily reflected in larger relaxation enthalpies [9]. However, understanding the behavior of LPBF BMGs at elevated temperatures is of importance because they could be post-processed via thermoplastic forming (TPF) [18,19,20]. During TPF, BMGs are heated to temperatures within the supercooled liquid (SCL) region, then shaped and cooled fast enough that they re-vitrify [18,21]. The SCL region is defined as the temperature range between the glass transition temperature, where the BMG transforms into a supercooled liquid, and the crystallization temperature [20,22,23]. Thus, BMGs can be processed similarly to plastics [18]. Once the glass transition sets in, the viscosity drastically drops, allowing for near-net shaping of the supercooled liquid into complex geometries and, especially, defined surface topographies [20,24,25]. Post-processing of additively manufactured BMGs via TPF could further densify them and reduce their surface roughness, a deficiency common to all metallic components fabricated by AM techniques [7,26,27].

The present work aims to extend the understanding of the behavior of additively manufactured BMGs at elevated temperatures. To achieve this purpose, we studied the temperature dependence of viscosity for the Zr_52.5_Cu_17.9_Ni_14.6_Al_10_Ti_5_ BMG synthesized by LPBF and copper mold casting. Investigating the viscous flow is of importance because LPBF allows to prepare large BMGs of marginal glass-forming alloys. They can be subsequently post-processed by thermoplastic forming or embossing [19,24,28]. Here, for the first time, we carried out preliminary tests of thermoplastic embossing of additively manufactured BMGs. Since cast BMGs have been intensively studied at elevated temperatures within the last decades, this work answers the question as to whether this gained knowledge can be transferred to additively manufactured BMGs.

## 2. Materials and Methods

Pre-alloyed ingots with nominal composition Zr_52.5_Cu_17.9_Ni_14.6_Al_10_Ti_5_ (at.%) were prepared from pure elements (purity > 99.9 wt.%) in an arc melting device (Edmund Bühler GmbH, Bodelshausen, Germany) in a Ti-gettered Ar atmosphere. Each ingot was re-melted four times to ensure chemical homogeneity. Subsequently, all ingots were gas-atomized by Nanoval GmbH & Co. KG (Berlin, Germany) via electrode induction-melting gas atomization (EIGA). The particle size distribution was determined via dynamic image analysis (CAMSIZER X2, Retsch TECHNOLOGY, Haan, Germany). After sieving, the powder had a particle size below 90 µm. Afterwards, it was processed via LPBF in an Ar-purged SLM 50 system (Realizer GmbH, Borchen, Germany) equipped with a fiber laser (spot size: 50 µm) to fabricate cylindrical samples with a diameter of 3 mm and height of 8 mm, as well as rectangular block specimens (5 × 4 × 2 mm^3^). Thereby, a laser power of 109.5 W, scanning velocity of 1000 mm/s, layer thickness of 40 µm, and hatch distance of 200 µm were employed. Unidirectional vectors were used and rotated by 90° in neighboring layers. In addition, rods with a diameter of 3 mm and length of 55 mm were prepared by suction casting in an arc melter (Edmund Bühler GmbH, Bodelshausen, Germany), and a plate (35 × 40 × 1.7 mm^3^) was synthesized by copper mold casting. Rectangular specimens (5 × 5 × 1.7 mm^3^) were prepared from this plate by wire erosion. The density of the LPBF BMG samples was determined according to the Archimedean principle using a Sartorius balance (MSA 225S, Göttingen, Germany). Structural characterization was carried out by X-ray diffraction (XRD) with a STOE STADI P diffractometer (STOE & Cie GmbH, Darmstadt, Germany) utilizing Mo–K_α1_ radiation (= 0.07093187 nm) in transmission mode. A step size of 0.7° and measuring duration of 100 s per step were employed. For quantifying the first broad maximum (13–22° (2θ)), longer measurements with a step size of 0.1° were conducted. Additionally, the microstructure of the LPBF-BMG specimens was observed in a scanning electron microscope (SEM, Gemini 1530, Carl ZeissAG, Oberkochen, Germany). Differential scanning calorimetry (DSC) was conducted in a Perkin Elmer Diamond at 20 K/min up to 873 K in Al crucibles. The analysis “Pyris series” was used to determine the characteristic temperatures and enthalpies. Thereby, *T*_g_ and *T*_x_ indicate the onset temperatures of glass transition and crystallization, respectively. The enthalpies represent the enclosed area by the curve and the respective limits characterizing the beginning and end of the event. The viscosity of the supercooled liquid as a function of the temperature was measured isochronally between 298 and 873 K by parallel plate rheometry (heating rate 20 K/min; constant load 2.6 N; cylindrical intender with a diameter of 3 mm) using a Perkin-Elmer TMA7 thermal mechanical analyzer (Waltham, MA, USA) operating under a high-purity argon atmosphere. Particular care was taken to ensure that indenter and specimens had the same diameter (3 mm) and that the different specimens had the same height (*h* = 175 ± 10 µm). The surfaces of the corresponding specimens were grinded and polished prior to testing. Moreover, preliminary thermoplastic embossing tests were carried out at 4 N using specimens with dimensions of 5 × 5 × 0.6 mm^3^ for the cast and 4 × 5 × 0.6 mm^3^ for the LPBF specimens. The topography resulting from thermoplastic embossing was investigated using a MicroProf optical profilometer equipped with a CHR150N Sensor (Fries Research & Technology GmbH, Bergisch Gladbach, Germany). The analysis software FRT Mark III Version 3.6 was used to analyze the topographical data. The oxygen contents of the LPBF and cast specimens, measured using carrier gas hot extraction (TC-436DR, LECO, St. Joseph, MI, USA), were 920 and 80 ppm, respectively.

## 3. Results and Discussion

**Figure 1:**
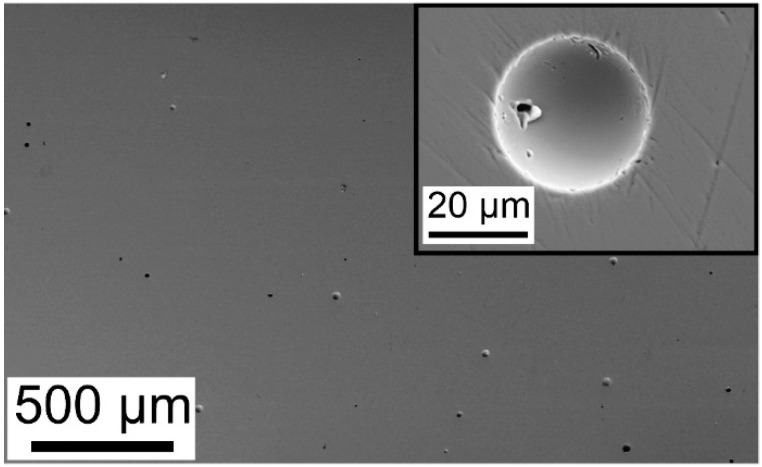
Secondary electron micrograph of the cross-section of the LPBF bulk metallic glass (BMG). Multiple pores can be observed.

The structure of BMGs synthesized by LPBF exhibited multiple pores, which were distributed across the specimen in a uniform manner (Figure 1).

As pointed out by several works, the evolution of pores is inevitable during LPBF [8,9,10,11,12,13,14,15,16,17]. It can arise during the powder deposition, from hollow powder particles and from the laser power distribution [8]. The relative density of the present LPBF-BMG samples was 98.7 ± 0.04%. It was measured using the Archimedean method and calculated with respect to the density of the corresponding cast BMG. A more detailed characterization of the structure and the thermal stability of the almost fully dense Zr_52.5_Ti_5_Cu_18_Ni_14.5_Al_10_ BMG fabricated by LPBF have been already reported elsewhere [8,9]; however, in order to put the present work into the proper perspective, the main characteristics are reported here as well.

**Figure 2:**
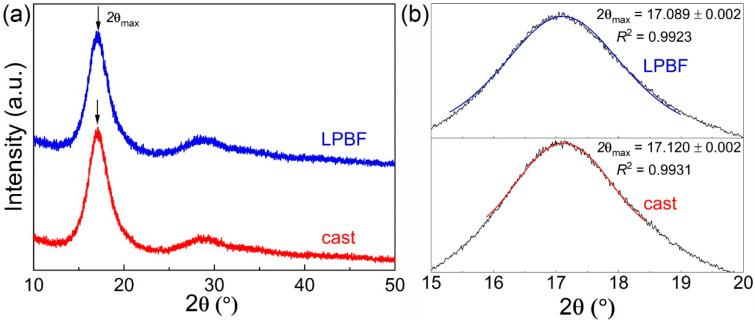
(**a**) Representative XRD patterns for the Zr_52.5_Ti_5_Cu_18_Ni_14.5_Al_10_ BMGs fabricated by casting and LPBF exhibiting the broad diffraction peaks characteristic of amorphous materials. (**b**) Position of the main scattering peak (2θ_max_) evaluated by fitting using a Pseudo-Voight function.

The Zr_52.5_Ti_5_Cu_18_Ni_14.5_Al_10_ specimens synthesized by casting and LPBF were both amorphous, as demonstrated by the XRD patterns in Figure 2a, which exhibits the broad diffraction peaks characteristic of amorphous materials and no trace of sharp reflections corresponding to crystalline phases.

However, the resolution of XRD was not sufficient to detect a limited volume fraction of nanocrystals. Therefore, transmission electron microscopy (TEM) was carried out in a previous work [8] for LPBF-BMG samples synthesized at identical conditions from the identical powder. The representative bright-field TEM image displayed a homogeneous maze-like pattern, and the corresponding selected area diffraction (SAED) pattern only exhibited broad and diffuse rings. The TEM results strongly indicate that the LPBF-BMG sample is also fully amorphous at the nanoscale. From the XRD pattern, the positions of the main scattering peak (2θ_max_ in Figure 2a) are inferred, and they can be used to examine the structural changes taking place in the medium-range order of metallic glasses [29]; they are 17.120 ± 0.002° and 17.089 ± 0.002° for the samples fabricated by casting and LPBF, respectively (Figure 2b). This discrepancy can be ascribed to a larger free volume [29,30] or higher oxygen content [15,31] in the LPBF material. At the moment, we cannot distinguish between the two effects, as the separation of free volume and oxygen contributions would require the synthesis of specimens with controlled free volume and oxygen contents combined with in situ high-energy XRD measurements during heating [29].

**Figure 3:**
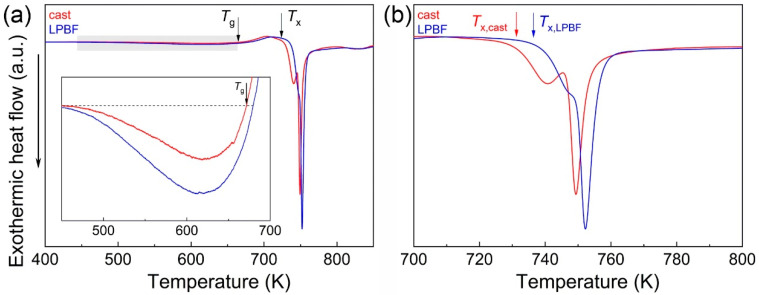
Isochronal DSC curves (heating rate 20 K/min) for the Zr_52.5_Ti_5_Cu_18_Ni_14.5_Al_10_ BMGs fabricated by casting and LPBF. (**a**) The grey area indicates the temperature range where structural relaxation takes place. *T_g_* and *T_x_* mark the temperatures of glass transition and crystallization. Inset: magnified segment of the DSC curve highlighting the effect of structural relaxation. (**b**) Magnified temperature section in which crystallization occurs. The crystallization onset temperature of the LPBF-BMG sample is higher, most likely due to the higher oxygen content.

Figure 3a shows the isochronal (20 K/min) DSC scans of the Zr_52.5_Ti_5_Cu_18_Ni_14.5_Al_10_ specimens synthesized by casting and LPBF. The DSC curves exhibited distinctive features characteristic of metallic glasses. At low temperatures, the curves displayed a broad exothermic event related to structural relaxation (indicated by the grey area in Figure 3a), where free volume is annihilated [32].

An endothermic effect due to the glass transition (*T_g_*) occurs at higher temperatures, followed by two overlapping exothermic events with onset temperature *T_x_* due to crystallization of the glass. The specimens display minor differences in their DSC curves (see Table 1). For example, the LPBF glass shows stronger structural relaxation (inset in Figure 3a), which can be ascribed to a higher free volume content, in agreement with the XRD results in Figure 2b, and higher crystallization temperature (Figure 3b and Table 1), most likely related to the higher oxygen content [31]; nevertheless, the cast and LPBF specimens exhibited a remarkably similar overall thermal stability despite the drastically different methods of materials synthesis.

The supercooled liquid (SCL) region, Δ*T_x_*, defined as the difference between *T_x_* and *T_g_*, is the temperature range where the viscosity is reduced by several orders of magnitude, a phenomenon of primary importance for thermoplastic forming of metallic glasses [19,24,28]. Accordingly, we investigated the variation of viscosity within the SCL region for the Zr_52.5_Ti_5_Cu_18_Ni_14.5_Al_10_ specimens synthesized by casting and LPBF using parallel plate rheometry measurements.

**Figure 4:**
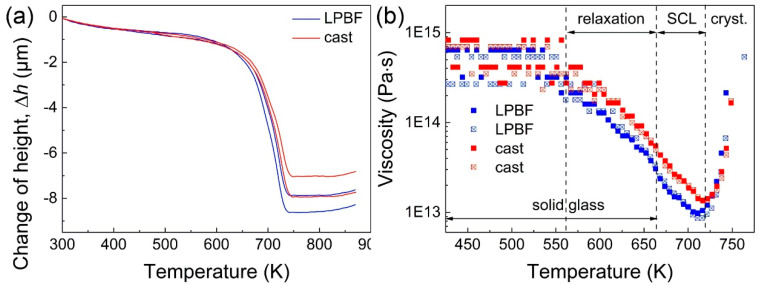
(**a**) Change of sample height (Δ*h*) as a function of temperature for the cast and LPBF specimens obtained by parallel plate rheometry and (**b**) corresponding temperature dependence of the viscosity of the supercooled liquid (SCL) derived using Equation (1). The characteristic temperature ranges where structural relaxation and crystallization occur are shown in (**b**) along with the range where the glassy solid transforms into the SCL.

Figure 4a shows the change of sample height (Δ*h*) vs. temperature for the cast and LPBF specimens obtained by parallel plate rheometry. The curves exhibited the same overall behavior, namely a limited decrease in height at temperatures below about 553 K, followed by a steeper decrease at high temperatures, before a final stage where the sample height does not change anymore at increasing temperatures. Similar to the DSC scans (Figure 3), the curves in Figure 4a are remarkably comparable, showing a final reduction of height of 8 ± 1 µm, with the difference between the specimens most likely due to the different porosity in the LPBF material (1.3% [9]).

The viscosity η as a function of temperature was then derived from the change of the sample’s height using Stefan’s equation [33,34]

(1) $$\eta =\frac{2Fh^{3}}{3\pi a^{4}\left(dh/dt\right)},$$

where *F* is the applied load, *a* is the radius of the plates, and *h* is the height of the sample. Figure 4b shows the temperature dependence of the viscosity for the cast and LPBF specimens derived using Equation (1). At low temperatures below 553 K, the viscosity was rather constant, and the glassy samples behaved as rigid objects. This initial stage was followed by a strong reduction of viscosity characterized by two distinct slopes. The first reduction occurred in the range 553–663 K, where the viscosity decreased relatively slowly. This behavior can be ascribed to structural relaxation and to annihilation of excess free volume [35] and, as such, it should be considered a densification phenomenon rather than softening. The second reduction occurred between 663 and 715 K, where the decrease in viscosity was more abrupt than the previous relaxation step, compatible with the viscous flow behavior observed in metallic glasses above *T_g_* [34]. Finally, at temperatures exceeding 715 K, the viscosity increased abruptly due to the crystallization, and the material lost its liquid-like behavior [34].

The rheometry results in Figure 4 allow us to identify the effective temperature range where the material behaves as a SCL; this range (Δ*T_x_* = *T_x_* − *T_g_* = 715 − 663 K) was different compared to the range identified by DSC (Δ*T_x_* = *T_x_* − *T_g_* = 746 − 686 K), indicating that the optimal experimental parameters for embossing of BMGs should be identified using viscosity data. In analogy with the data in Figure 3, the viscosity curves of the cast and LPBF specimens displayed minor differences. For example, the minimum viscosity characterizing the LPBF material was lower than the cast counterpart. This aspect might suggest stronger softening in the LPBF specimen that might then facilitate embossing at given processing conditions. However, this difference can also be ascribed to the higher porosity of the LPBF glass; at temperatures within the SCL region, the low viscosity may induce easy collapsing of the pores, leading to material densification rather than contributing to a real reduction of viscosity. To clarify this aspect, we performed preliminary thermoplastic embossing tests using parallel plate rheometry with samples larger than the indenter (3 mm).

**Figure 5:**
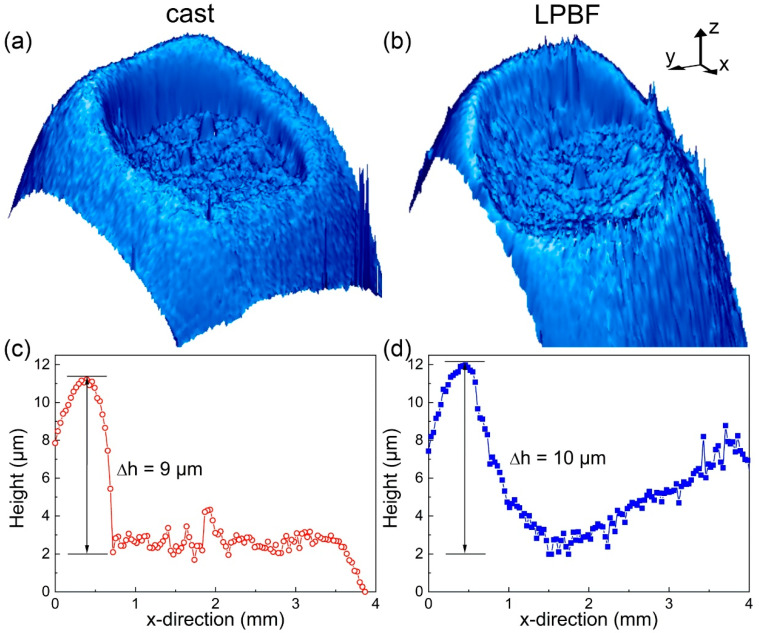
Topographies of the Zr_52.5_Ti_5_Cu_18_Ni_14.5_Al_10_ BMGs fabricated by (**a**) casting and (**b**) LPBF after thermoplastic embossing. Depth profiles along the x-direction quantifying the sharpness of the indentations in the BMGs fabricated by (**c**) casting and (**d**) LPBF.

Figure 5a,b show the topography of the cast and LPBF BMGs after thermoplastic embossing. The shape of the indentations originates from the geometry of the cylindrical intender used for parallel plate rheometry, so they extend to about 3 mm in diameter.

Although the device for parallel plate rheometry is not optimized for embossing experiments, the indentations were clearly visible on the surface of both specimens and were remarkably similar. The indentations were not regular, exhibiting a pronounced edge characterized by a steep height reduction. The indentation of the BMG fabricated by casting displayed a sharper depth profile, where the reduction amounted to 9 µm and occurred within about 250 µm (Figure 5c). The indentation on the LPBF BMG was less sharp because a similar height reduction (10 µm) was visible over a wider range of almost 500 µm (Figure 5d). Owing to the smaller area of the LPBF BMG specimen, the positioning of the intender at the sample center is more challenging. In the present experiment, the positioning was slightly off, which resulted in a marginal sideways motion of the intender during thermoplastic embossing, so that the indent was less sharp. Microstructural characterization of the LPBF specimen after thermoplastic embossing could confirm whether the inherent porosity might be the origin of its stronger softening (Figure 4). As the LPBF BMG was heated and transformed into a supercooled liquid, the lower viscosity allowed for thermoplastic forming, so that the pores also should change in shape and size until they collapsed. However, with the present setup (parallel plate rheometry) used for preliminary thermoplastic embossing tests, only low forces up to 4 N could be exerted, which led to a low height reduction of 10 µm. Since the structure of the LPBF BMG exhibited pores more than 30 µm in diameter (Figure 1, inset), distinctly distorted pores could not be observed (not shown here). Further experiments will be conducted at higher loading conditions to provide experimental proof.

Nevertheless, these preliminary results demonstrate the suitability of additively manufactured BMGs for thermoplastic forming and embossing. We believe that the knowledge gained from TPF of cast BMGs can be transferred to additively manufactured BMGs. Consequently, all TPF-based processing methods, such as compression and injection molding, hot-rolling, extrusion, blow molding, and micro- and nano-embossing, should be also applicable [18]. Another effect that should be considered when processing LPBF BMGs is the thermal embrittlement occurring during TPF of cast BMGs during subsequent cooling at slower rates [18]. Involved processes of structural relaxation are accompanied by significant changes in physical properties of the metallic glass, namely the severe embrittlement monitored via fracture toughness, impact toughness, and bending ductility measurements [36,37].

Lastly, we want to emphasize that particularly large BMGs can be fabricated by AM techniques and from marginal glass-forming compositions. Specific examples could be, for instance, biocompatible Mg- and Ti-based alloys not containing elements harmful to the human body, but in turn required for enhanced glass-forming ability [38,39,40,41]. Since TPF not only permits shaping the whole volume of the BMG specimen, but also solely embossing its surface, micro- to nanostructured patterns can be generated [24], which involve programming a desirable cell response [42]. Consequently, AM enables the functionalization of BMGs from marginal glass-forming alloys, and future work will be conducted to further explore this issue.

## 4. Conclusions

The temperature dependence of viscosity in Zr_52.5_Cu_17.9_Ni_14.6_Al_10_Ti_5_ bulk metallic glasses fabricated by copper mold casting and laser powder bed fusion (LPBF) has been investigated by parallel plate rheometry. The overall viscous flow behaviors in the specimens fabricated by the different techniques were rather similar, with minor differences that can be ascribed to the higher porosity of the LPBF metallic glass. Preliminary thermoplastic embossing tests revealed remarkably similar results in the specimens synthesized by LPBF and casting. The present findings have important implications for giving extra freedom in materials shaping and opening the way to surface functionalization by thermoplastic embossing of additively manufactured BMGs.

## Figures and Tables

**Table 1:** Overview of the characteristic temperatures of Zr_52.5_Ti_5_Cu_18_Ni_14.5_Al_10_ BMGs synthesized by LPBF and copper mold casting determined via DSC (heating rate 20 K/min). Glass transition (*T*_g_) and crystallization (*T*_x_) temperatures, the width of the supercooled liquid region (Δ*T*_x_), relaxation enthalpies (Δ*H*_relax_), and crystallization enthalpies (Δ*H*_cryst_) are listed.

	*T*_g_ (K)	*T*_x_ (K)	Δ*T*_x_ (K)	Δ*H*_relax_ (J/g)	Δ*H*_cryst_ (J/g)
cast1	684	748	64	−3.9	−85.2
cast2	689	745	56	−1.4	−85.2
cast3	686	746	61	−3.7	−85.8
cast_ave_	686 ± 2.7	746 ± 1.2	60 ± 3.9	−3.0 ± 1.40	−85.4 ± 0.38
LPBF1	686	749	63	−11.7	−81.6
LPBF2	689	748	59	−12.6	−78.7
LPBF3	688	750	62	−7.8	−78.8
LPBF_ave_	688 ± 1.7	749 ± 0.8	61 ± 2.1	−10.7 ± 2.55	−79.7 ± 1.62
